# Recognition of the SARS-CoV-2 receptor binding domain by neutralizing antibodies

**DOI:** 10.1016/j.bbrc.2020.10.012

**Published:** 2021-01-29

**Authors:** Meng Yuan, Hejun Liu, Nicholas C. Wu, Ian A. Wilson

**Affiliations:** a Department of Integrative Structural and Computational Biology, The Scripps Research Institute, La Jolla, CA, 92037, USA; b Department of Biochemistry, University of Illinois at Urbana-Champaign, Urbana, IL, 61801, USA; c Carl R. Woese Institute for Genomic Biology, University of Illinois at Urbana-Champaign, Urbana, IL, 61801, USA; d Center for Biophysics and Quantitative Biology, University of Illinois at Urbana-Champaign, Urbana, IL, 61801, USA; e IAVI Neutralizing Antibody Center and the Collaboration for AIDS Vaccine Discovery (CAVD), The Scripps Research Institute, La Jolla, CA, 92037, USA; f Consortium for HIV/AIDS Vaccine Development (CHAVD), The Scripps Research Institute, La Jolla, CA, 92037, USA; g The Skaggs Institute for Chemical Biology, The Scripps Research Institute, La Jolla, CA, 92037, USA

**Keywords:** SARS-CoV-2, SARS-CoV, Neutralizing antibodies, Receptor binding domain (RBD), RBD natural mutations, Epitopes, Germline-encoded motifs, Cross-neutralization, Antibody avidity

## Abstract

Immediately from the outset of the COVID-19 pandemic, researchers from diverse biomedical and biological disciplines have united to study the novel pandemic virus, SARS-CoV-2. The antibody response to SARS-CoV-2 has been a major focus of COVID-19 research due to its clinical relevance and importance in vaccine and therapeutic development. Isolation and characterization of antibodies to SARS-CoV-2 have been accumulating at an unprecedented pace. Most of the SARS-CoV-2 neutralizing antibodies to date target the spike (S) protein receptor binding domain (RBD), which engages the host receptor ACE2 for viral entry. Here we review the binding sites and molecular features of monoclonal antibodies that target the SARS-CoV-2 RBD, including a few that also cross-neutralize SARS-CoV.

## Highlights

• The receptor binding domain is a major target of antibodies to SARS-CoV-2.
• Neutralizing antibodies target various epitopes on the receptor binding domain.
• Convergent antibody responses to SARS-CoV-2 are observed.
• Cross-neutralization of SARS-CoV-2 and SARS-CoV maps to two RBD epitopes.
• Antibody avidity can be crucial for neutralization potency against SARS-CoV-2.

## 1 Introduction

Coronavirus disease 2019 (COVID-19) is an ongoing pandemic caused by severe acute respiratory syndrome coronavirus 2 (SARS-CoV-2). The first known COVID-19 case was identified in December 2019 [1] and SARS-CoV-2 has now spread world-wide to at least 188 countries/regions (https://coronavirus.jhu.edu/map.html). As of October 2, 2020, over 34 million cases and over 1 million deaths have been reported (www.who.int). The COVID-19 pandemic has affected global health, as well as society in general, including the economy, schools, and differential effects on demographics. Thus, effective and safe vaccines and therapeutics are urgently needed.

**Fig. 1:**
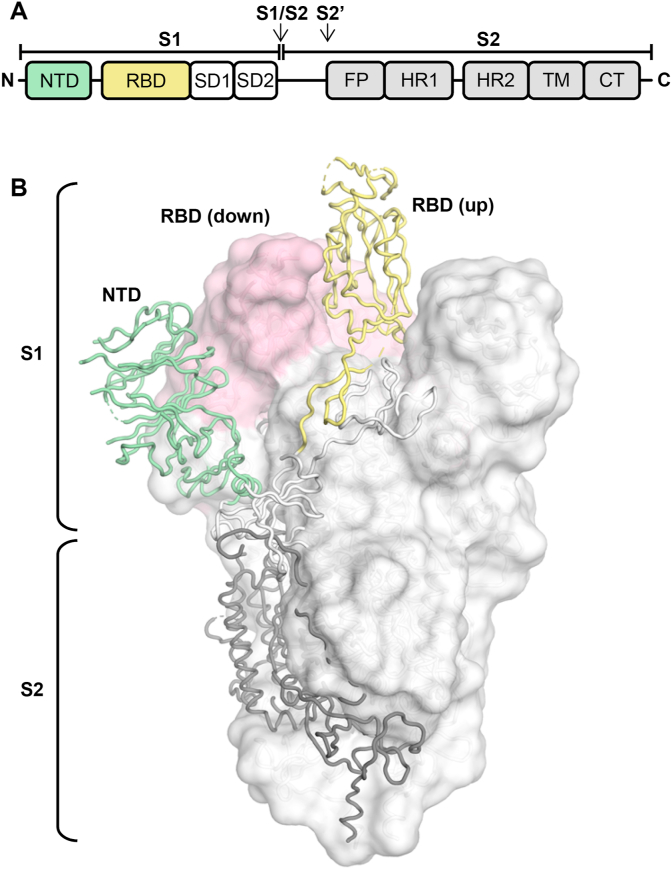
**Schematic and structure of the SARS-CoV-2 spike (S) protein. (A)** Schematic of the SARS-CoV-2 S protein. The receptor binding domain (RBD) is colored in yellow. NTD: N-terminal domain; SD: subdomain (1 and 2); FP, fusion peptide; HR1, heptad repeat 1; HR2, heptad repeat 2; TM, transmembrane domain; CT, cytoplasmic tail. Arrows denote protease cleavage sites. **(B)** Structure of the SARS-CoV-2 S protein in the prefusion conformation with one RBD in an up conformation and two RBDs in the down conformation (PDB ID: 6VSB) [46]. For the S1 domain, RBD (in an up conformation) and NTD are highlighted in yellow and green, respectively, with the remainder of S1 and S2 shown in white and grey. For clarity, only one protomer is represented in tubes and the other two subunits are represented by a white molecular surface with their RBDs in a down conformation in pink.

SARS-CoV-2 is an enveloped, positive-strand RNA virus in the betacoronavirus genus [2]. Other betacoronavirus strains that have infected humans include SARS-CoV, MERS-CoV and the seasonal CoVs, HCoV-OC43 and HCoV-HKU1. SARS-CoV-2 is genetically related to SARS-CoV (∼80% sequence identity at the nucleotide level [3]), which caused an epidemic in 2003 with more than 8000 cases in 26 countries (www.who.int). Like SARS-CoV, SARS-CoV-2 binds to the host receptor, angiotensin-converting enzyme 2 (ACE2), through the receptor binding domain (RBD) of its spike (S) glycoprotein to mediate cell entry [3]. The S protein is present as a homotrimer on the virus, with its S1 head domain perched atop the S2 stalk, which is involved in membrane-fusion. Two proteolytical cleavage sites are present at the S1/S2 boundary and in S2 immediately before the fusion peptide (Fig. 1A). The S1 domain contains an N-terminal domain (NTD) and the RBD that connects to two subdomains (SD1, SD2) at the C-terminus. The RBD transiently switches between an up state and a down state (Fig. 1B) and can only bind to ACE2 in the up state, since the receptor binding site (RBS) is partially buried in the down state.

Molecular and functional understanding of the antibody response against SARS-CoV-2 can then provide insights into vaccine and therapeutic design. Here we provide an overview of the molecular features of antibody recognition of the SARS-CoV-2 RBD. Three major epitopes on the RBD are recognized by the currently characterized SARS-CoV-2 neutralizing antibodies. Most of these antibodies are specific to SARS-CoV-2, but a few can also cross-react between SARS-CoV-2 and SARS-CoV. We also highlight the importance of avidity in antibody neutralization, as well as the potential emergence of antibody escape mutants.

## 2 Antibodies to the SARS-CoV-2 RBD

The RBD of the SARS-CoV-2 S protein is a major target for the antibody response [4]. As of October 2, 2020, more than 800 SARS-CoV-2-targeting monoclonal antibodies from COVID-19 patients have been disclosed in 20 different studies [[5], [6], [7], [8], [9], [10], [11], [12], [13], [14], [15], [16], [17], [18], [19], [20], [21], [22], [23], [24]]. 486 of 822 have been reported to target the RBD, 290 of which have measurable neutralization activity [[5], [6], [7], [8], [9], [10], [11], [12], [13], [14], [15], [16], [17], [18], [20], [21], [22], [23], [24]] (n.b. information on these antibodies was obtained from CoV-AbDab [25] from the version on October 2, 2020).

**Fig. 2:**
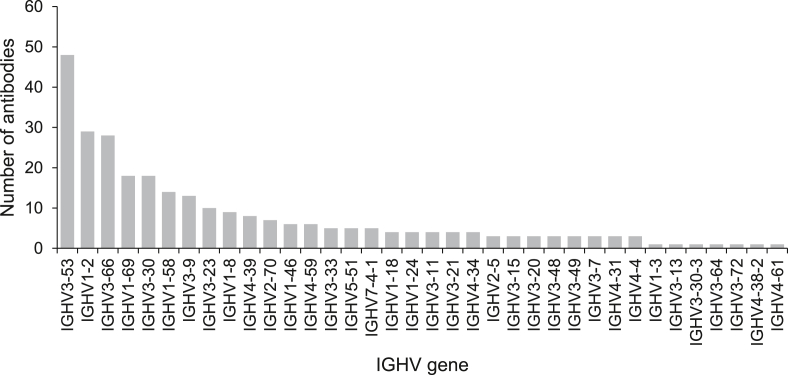
**The distribution of IGHV gene usage of RBD-targeting neutralizing antibodies.** A total of 280 antibodies reported in 19 studies are analyzed [[5], [6], [7], [8], [9], [10], [11], [12], [13], [14], [15], [16], [17], [18], [20], [21], [22], [23], [24]].

**Fig. 3:**
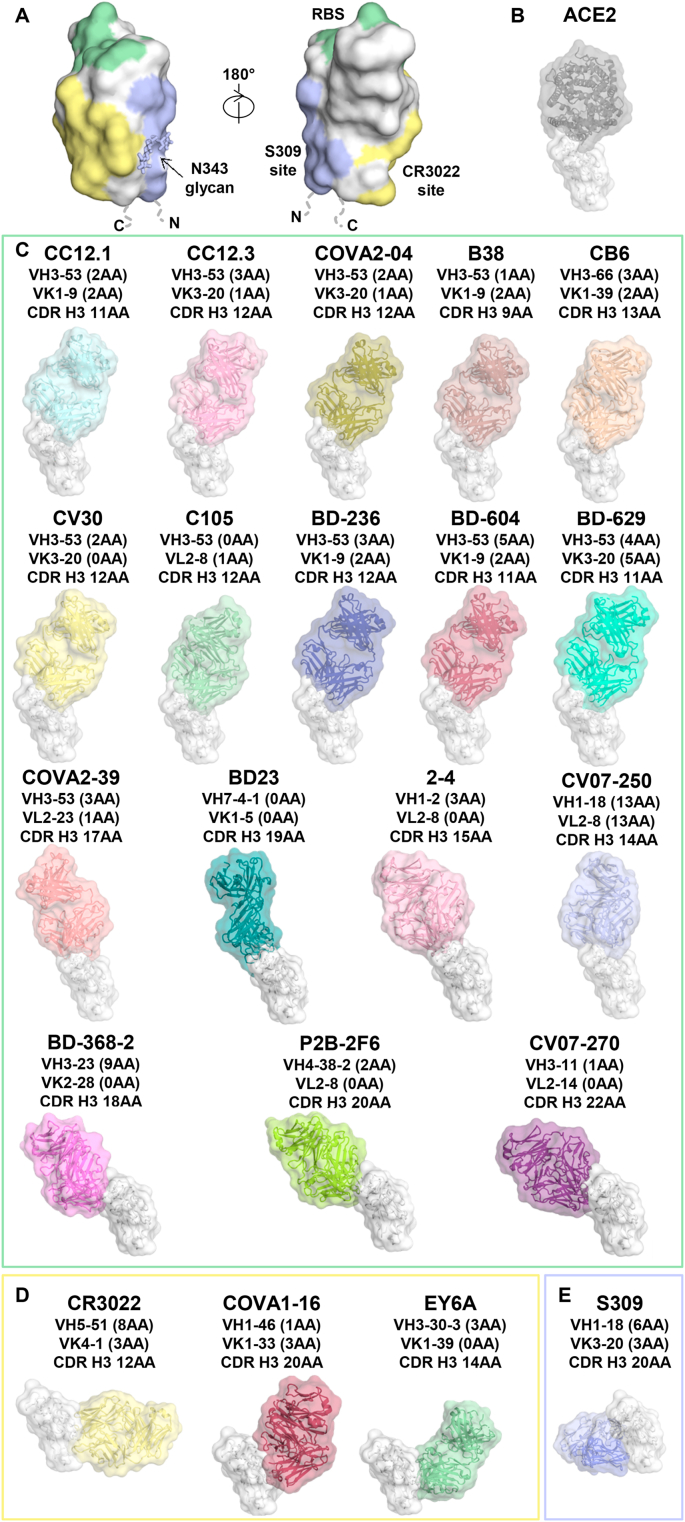
**Antibody recognition of the RBD of SARS-CoV-2. (A)** The main antigenic sites on the RBD of SARS-CoV-2 that have been identified to date. The RBD is shown in white, with the receptor-binding site (RBS) in green, CR3022 epitope in yellow, and S309 epitope in blue. **(B**–**E)** Structures of the SARS-CoV-2 RBD with **(B)** human ACE2 receptor (PDB ID: 6M0J) [34], and **(C**–**E)** neutralizing antibodies isolated from human patients. The RBDs (white) are all shown in the same relative orientation. Numbers of somatic mutated residues are shown in brackets after the light and heavy chain germline designation. The length of CDR H3 is indicated using the IMGT definition. **(C)** Structures of the RBD in complex with RBS-targeting antibodies: CC12.1 (PDB ID: 6XC2) [27], CC12.3 (PDB ID: 6XC4) [27], COVA2-04 (PDB ID: 7JMO) [28], B38 (PDB ID: 7BZ5) [23], CB6 (PDB ID: 7C01) [13], CV30 (PDB ID: 6XE1) [29], C105 (PDB ID: 6XCM) [30], BD-236 (PDB ID: 7CHB) [7], BD-604 (PDB ID: 7CH4) [7], BD-629 (PDB ID: 7CH5) [7], COVA2-39 (PDB ID: 7JMP) [28], BD23 (PDB ID: 7BYR) [16], 2–4 (PDB ID: 6XEY) [15], CV07-250 (PDB ID: 6XKQ) [6], BD-368-2 (PDB ID: 7CHE) [7], P2B-2F6 (PDB ID: 7BWJ) [17], and CV07-270 (PDB ID: 6XKP) [6]. **(D)** Structures of the RBD in complex with antibodies targeting the CR3022-binding site: CR3022 (PDB ID: 6W41) [31], COVA1-16 (PDB ID: 7JMW) [32], and EY6A (PDB ID: 6ZER) [14]. **(E)** Structure of antibody S309 with the RBD (PDB ID: 6WPT) [33].

Various germline genes are used in RBD-targeting neutralizing antibodies. Here we summarize the V genes of neutralizing antibodies isolated from COVID-19 patients (Fig. 2, Fig. 3). The germline gene usage shows a strong preference for IGHV3-53, IGHV1-2, and IGHV3-66^1^ (Fig. 2). In comparison, the frequencies of these V_H_ genes only rank 20, 23, and 26 in healthy individuals, respectively [26], suggesting that certain germline genes are naturally favored for targeting the RBD. Furthermore, if we combine the highly related IGHV3-53 and IGHV3-66 germlines (which only differ by one amino acid), this gene family is even more dominant compared to other heavy chain genes.

Extensive efforts have been made on understanding antibody recognition of the RBD at the molecular level. As of October 2, 2020, structures of 22 different human antibodies in complex with the SARS-CoV-2 spike were available in the Protein Data Bank (PDB) (Fig. 3) [6,7,[12], [13], [14], [15], [16], [17],^23^,[27], [28], [29], [30], [31], [32], [33]]. These antibodies can be clustered based on their interaction with three distinct RBD binding sites: (1) receptor binding site; (2) CR3022 cryptic site; and (3) S309 proteoglycan site (Fig. 3A). Most of the antibodies that bind to these three sites show neutralizing activity with SARS-CoV-2. Moreover, antibodies that bind the CR3022 and S309 sites have a tendency to cross-react with SARS-CoV RBD, as compared to antibodies to the RBS, which are highly specific for SARS-CoV-2.

## 3 Neutralizing antibodies targeting the receptor-binding site

A previous study showed that 17 residues of the SARS-CoV-2 RBD make direct contact with human ACE2, with an interface area of 863 Å^2^ (Fig. 3B and Table 1) [34]. Only eight out of these 17 residues are conserved between SARS-CoV-2 and SARS-CoV [34]. Structural studies have been reported for 17 human antibodies that target the receptor-binding site (RBS): CC12.1, CC12.3 [27], COVA2-04, COVA2-39 [28], B38 [23], CB6 [13], CV30 [29], C105 [30], BD-236, BD-604, BD-629, BD-368-2 [7], BD23 [16], 2–4 [15], CV07-250, CV07-270 [6], and P2B-2F6 [17]. Since the epitopes of these 17 antibodies largely overlap with the ACE2-binding site (the epitope is therefore named here as “RBS”), they can neutralize SARS-CoV-2 by competing with ACE2 binding at the RBS; the complete RBS epitope is only accessible when the RBD is in up conformation. However, these antibodies can approach and bind the RBD at different angles and, hence, overlap to different extents with the ACE2 binding site. We can sub-divide the current RBS antibodies into three different subgroups (A-C), where binding can be classified with respect to their angle of approach to the RBD and their relative disposition with respect to the ridge on the RBS. These antibodies vary considerably in their epitope size ranging from 626 Å^2^ to 1248 Å^2^ in buried surface area (BSA) (Table 1). The heavy chain also tends to dominate binding as in most antibodies. The RBS-A antibodies have the largest epitope both in BSA (863–1248 Å^2^) and residues contacted (19–35) or buried (30–40). In comparison, the epitopes of RBS-B antibodies [BSA (744–966 Å^2^), residues contacted (12–20) or buried (20–26)] and RBS-C antibodies [BSA (626–803 Å^2^), residues contacted (9–20) or buried (19–25)] are smaller, although the number of antibodies to these epitopes is limited at present (Table 1).

**Table 1:** Buried surface area (BSA) of epitopes and number of epitope residues.

	Antibody or receptor	BSA (Å^2^)^a^			Number of contact residues^b^	Number of buried residues^c^
		Overall	HC	LC		
**RBS-A**	CC12.1	1248	692	556	29	38
	B38	1193	689	504	35	40
	COVA2-04	1125	755	370	31	32
	BD-604	1106	712	394	30	36
	BD-236	1083	656	427	31	35
	CB6	1049	736	313	27	36
	BD-629	1015	822	193	25	30
	CV30	981	712	269	23	31
	C105	896	638	258	19	33
	CC12.3	863	677	186	24	30
**RBS-B**	CV07-250	966	422	543	20	26
	2–4	795	662	132	12	20
	BD23	765	731	34	14	22
	COVA2-39	744	603	141	17	21
**RBS-C**	CV07-270	803	696	108	20	25
	BD-368-2	699	569	130	9	19
	P2B-2F6	626	482	144	13	20
**ACE2**		863			17	26

a BSA of epitope residues was calculated using PISA program [83] for the sum of the surface area buried by the heavy (HC) and light chains (LC) as well as the individual heavy and light chain contributions.

b Epitope residues that contact antibody within a distance of 4 Å in the spike/RBD-antibody complex as calculated by the PISA program [83].

c Epitope residues with a BSA >0 Å^2^ as calculated by the PISA program [83].

### 3.1 Antibodies to RBS-A

The RBS-A antibodies to the SARS-CoV-2 RBD adopt a very similar binding angle to each other and the RBS-A epitope overlaps extensively with the ACE2-binding site (Fig. 3, Fig. 5B–C). In fact, 16 out of 17 residues in the ACE2-binding site are involved in binding to some of the RBS-A antibodies [27]. Similar to the ACE2-binding site, the RBS-A epitope is only accessible when the RBD is in the “up” conformation. Structures of 10 RBS-A antibodies have been reported so far: CC12.1, CC12.3 [27], COVA2-04 [28], B38 [23], CB6 [13], CV30 [29], C105 [30], BD-236, BD-604, and BD-629 [7] (Fig. 3C). Interestingly, all of these RBS-A antibodies are encoded by antibody germline genes IGHV3-53/3–66, albeit with different subsets of light chain genes, but still adopt a nearly identical angle of approach to the RBD. Further details about the RBS-A antibodies and their structural convergence are discussed in Section 3.4.

### 3.2 Antibodies to RBS-B

**Fig. 4:**
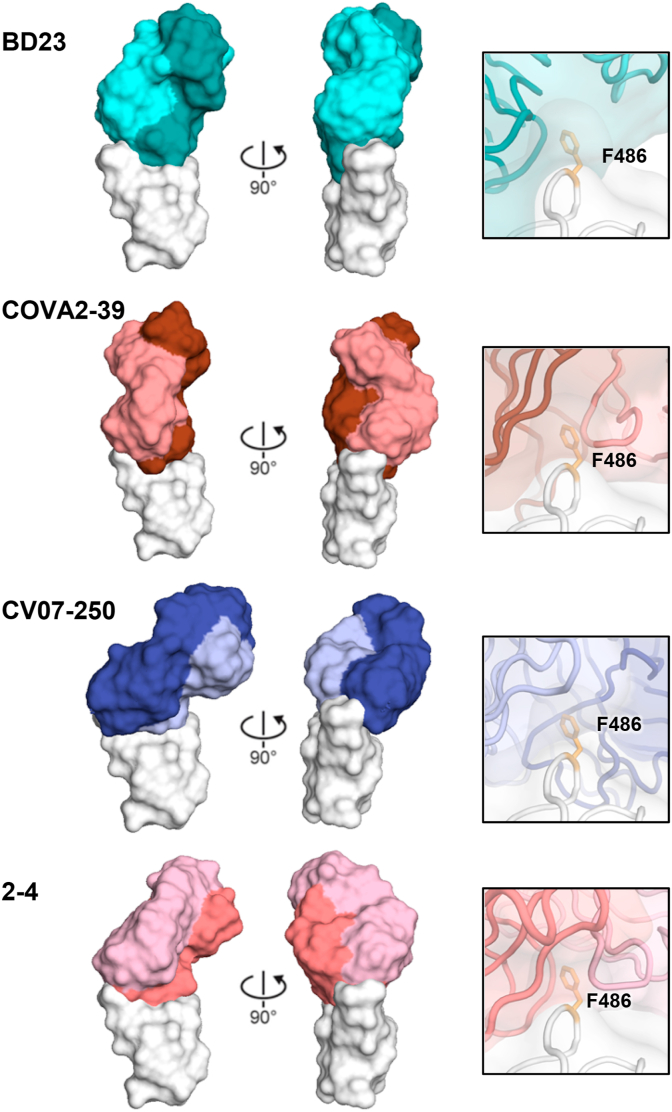
**Antibodies targeting the RBS-B epitope of SARS-CoV-2 RBD.** Structures of four RBS-B antibodies are shown: BD23 (PDB ID: 7BYR) [16], COVA2-39 (PDB ID: 7JMP) [28], CV07-250 (PDB ID: 6XKQ) [6], and 2–4 (PDB ID: 6XEY) [15]. The RBD is shown in white, and the heavy and light chains of the bound antibodies are shown in dark and light colors, respectively. These antibodies approach the RBD at different angles and with different relative rotations of the Fab on the RBD surface. Interaction between Phe486 of the RBD and each antibody is shown in the right panels, with Phe486 highlighted in orange sticks.

**Fig. 5:**
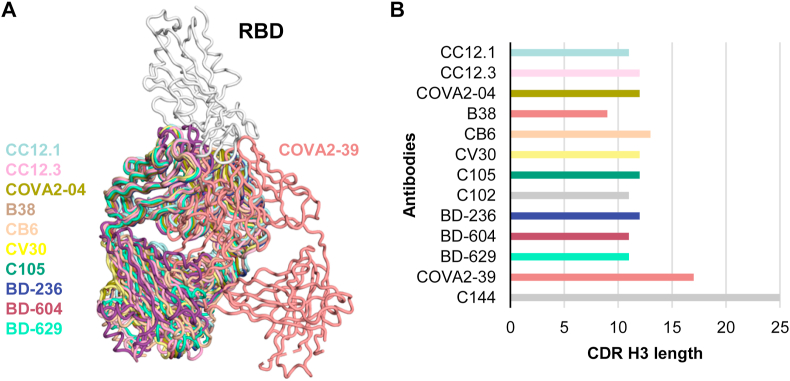
**Structural convergence of SARS-CoV-2 RBD-targeting antibodies encoded byIGHV3-53/3**–**66 genes. (A)** Comparison of two completely different binding modes of antibodies encoded by IGHV3-53/3–66 germline genes that target the SARS-CoV-2 RBD and have different CDR H3 lengths [short (mode A) and long (mode B)]. All structures were retrieved from the PDB and superimposed on their RBD (white). **(B)** CDR H3 length (IMGT numbering) of the antibodies is shown in the bar chart. The color coding of the antibodies is the same as in Fig. 3. Structures of two other SARS-CoV-2 RBD-targeting antibodies encoded by IGHV3-53/3–66, namely C102 and C144 [35], are not publicly available and are not included in this figure.

The binding sites of COVA2-39, BD23, 2–4, CV07-250 differ from RBS-A antibodies and are also encoded by different germline genes (Fig. 3C). They bind at a different angle and straddle the RBS ridge, where RBD Phe486 inserts into a pocket between the light and heavy chains of the Fab on the paratope surface (Fig. 4). They bind with more varied angles of approach than the RBS-A antibodies, where the Fabs can rotate around Phe486 on the epitope surface relative to each other [6,15,16,28] (Fig. 3, Fig. 5). Indeed, the Fab orientations differ by up to 90° and 180° relative to one another (Fig. 4). We will call these RBS-B antibodies to differentiate their epitope. The heavy and light chains from antibodies 2–4 and CV07-250 are both involved in recognition of the RBD, while BD23 almost exclusively binds the RBD with its heavy chain (Table 1). BD23 and 2–4 can bind to SARS-CoV-2 in the RBD down conformation, whereas binding of COVA2-39 and CV07-250 to the RBD in the down conformation would clash with the adjacent protomers of the spike trimer protein [6,15,16,28]. Furthermore, in contrast to RBD-targeting antibodies that typically carry low levels of somatic hypermutation (SHM), CV07-250 isolated 19 days after symptom onset had already acquired 33 SHM residues, including non-paratope SHM residues, suggesting that CV07-250 could have been initially affinity-matured against a different antigen [6].

### 3.3 Antibodies to RBS-C

The epitopes of three other antibodies, P2B-2F6 [17], CV07-270 [6], and BD-368-2 [7] are very similar to each other (Fig. 3C). These antibodies target the back side of the RBS on the opposite side of the RBS ridge, to an epitope we name as RBS-C, at a different angle of approach from those that target RBS-A and RBS-B. Antibodies that target RBS-C have fewer clashes with ACE2 binding to the RBD. Notably, these three antibodies targeting the RBS-C epitope use different germline genes from each other and from the other antibodies above. P2B-2F6 is encoded by IGHV4-38-2, CV07-270 by IGHV3-11, and BD-368-2 by IGHV3-23. Despite distinct germlines, the approach angles of these three antibodies to the RBD are very similar with similar Fab disposition on the epitope surface. These observations suggest that RBD-C is also highly immunogenic and can effectively elicit neutralizing antibodies. Furthermore, unlike ACE2 and RBS-A-targeting antibodies, which can only bind the RBD in an up conformation, structural studies have shown that RBS-C-targeting antibodies P2B-2F6, BD-368-2, and CV07-270 can bind both up and down RBD conformations [6,7,17].

### 3.4 Shared neutralizing antibodies to RBS-A and RBS-B encoded by IGHV3-53/3-66

IGHV3-53/3–66 genes are the most frequent genes used by antibodies targeting the SARS-CoV-2 spike protein and are shared among different people and in different studies [27]. In fact, within 290 of RBD-targeting neutralizing antibodies isolated so far, as reported in CoV-AbDab [25], 76 are encoded by these genes. Moreover, SARS-CoV-2 antibodies encoded by IGHV3-53/3–66 have low somatic hypermutation rates, but still achieve potent neutralization activity [27]. These observations suggest that the molecular features of IGHV3-53/3-66-encoded antibodies naturally favor recognition of the RBD [27]. Intriguingly, SARS-CoV-2 neutralizing antibodies encoded by IGHV3-53/3–66 generally have a shorter complementarity-determining region (CDR) H3 compared to non-IGHV3-53/3–66 antibodies, with a small subset containing a more conventional, longer CDR H3 [27,28,30,35].

Structures of 13 IGHV3-53/3-66-encoded antibodies have been determined, including eleven with short CDR H3 (length ≤ 13 AA, IMGT numbering) and two with long CDR H3 (≥17 AA, IMGT numbering) (Fig. 3, Fig. 5) [7,13,23,[27], [28], [29], [30]]. All eleven antibodies with short CDR H3 (CC12.1, CC12.3, COVA2-04, B38, CB6, CV30, C102, C105, BD-236, BD-604, and BD-629) exhibit almost identical angles of approach to the RBD (RBS-A), whereas COVA2-39 with a longer CDR H3 binds at a different angle to RBS-B. The epitopes of these antibodies overlap with the ACE2-binding site. Despite use of a common IGHV gene, different light-chain genes have been paired with IGHV3-53/3–66 and include IGKV1-9, IGKV3-20, IGKV1-39, and IGLV2-8 (Fig. 3C). Thus, IGHV3-53/3–66 seems to preferentially pair with distinct subsets of light chains to target the ACE2-binding site of the SARS-CoV-2 RBD. The CDR H3 length (short and long) also seems to correlate with which light chains are selected [28].

High-resolution structures have revealed key features of the antibodies that determine RBD recognition and binding mode. Based on structural analysis, the presence of two IGHV3-53/3–66 germline-encoded structural motifs, namely an NY motif in CDR H1 and an SGGS motif in CDR H2, as well as a short CDR H3, naturally favor RBD binding [27]. In contrast to IGHV3-53/3-66-encoded antibodies with short CDR H3 loops, COVA2-39 with a long CDR H3 still interacts with the RBS but in a very different binding mode (mode B) (Fig. 5) [28]. COVA2-39 is also rotated by about 180° around the Fab long axis compared to mode A (canonical mode, RBS-A) and contacts a different surface of the RBD (RBS-B) (Fig. 5A). Furthermore, another IGHV3-53-encoded antibody C144 with a long CDR H3 was recently reported to bind the RBS in a third mode (mode C) that is similar to mode B. However, in this binding mode, its CDR H3 (25 amino acids) can bridge between adjacent “down” RBDs to lock the S protein into a closed, prefusion conformation [35]. Nevertheless, the binding of these two antibodies still is strongly dependent on the structural motifs in the IGHV3-53/3-66-encoded CDR H1 and H2 even although the region of their RBD interaction is completely different from the corresponding IGHV3-53-encoded RBS-A antibodies [28,35]. Other SARS-CoV-2 neutralizing antibodies, such as 2–43 [15], C002, C121, C119 and C104 [35], also appear to simultaneously bind to two RBDs from adjacent protomers, indicating that quaternary interactions within the spike trimer are important for some antibodies.

## 4 Cross-neutralizing antibodies: CR3022 cryptic site

The S protein of SARS-CoV-2 is homologous to that of SARS-CoV, with amino-acid sequence identity of 77% for the S protein, and 73% for the RBD^2^ [36]. Notably, the receptor binding sites of the two viruses have diverged substantially from each other, with only eight out of 17 ACE2 receptor-binding residues being conserved [34]. Cross-reactive antibodies against the RBDs of both SARS-CoV and SARS-CoV-2 are relatively rare in the approximately 300 anti-SARS-CoV-2 RBD neutralizing antibodies according to the coronavirus antibody database CoV-AbDab [25]. Shortly after the COVID-19 outbreak, we and others reported that CR3022, an anti-SARS-CoV neutralizing antibody isolated from a SARS patient almost 15 years ago [37], exhibited cross-reactivity to SARS-CoV-2 RBD [31,[38], [39], [40]]. Despite its inability to neutralize SARS-CoV-2 virus infection [11,21,31,41], structural studies of CR3022 in complex with the RBD revealed a conserved cryptic site on the RBD that is vulnerable to cross-reactive antibody binding (Fig. 3D) [31,39,40]. Furthermore, we recently showed that a single mutation P384A fully determines the affinity and neutralization difference between SARS-CoV and SARS-CoV-2 [42].

Although additional cross-reactive antibodies have been reported [6,14,18,21,33,[43], [44], [45]], limited structural information is available on them. Two distinct epitopes have been found so far to be targeted by cross-reactive antibodies (Fig. 3D and E). The most frequent epitope targeted by cross-neutralizing antibodies to date is the CR3022 site, whereas the proteoglycan site that involves an N-glycan at N343 has been found to be recognized by a different cross-neutralizing antibody S309 [33]. It is nevertheless too early to evaluate the relative frequency of targeting these two different cross-neutralizing sites given the small number of antibodies characterized to these epitopes so far.

**Fig. 6:**
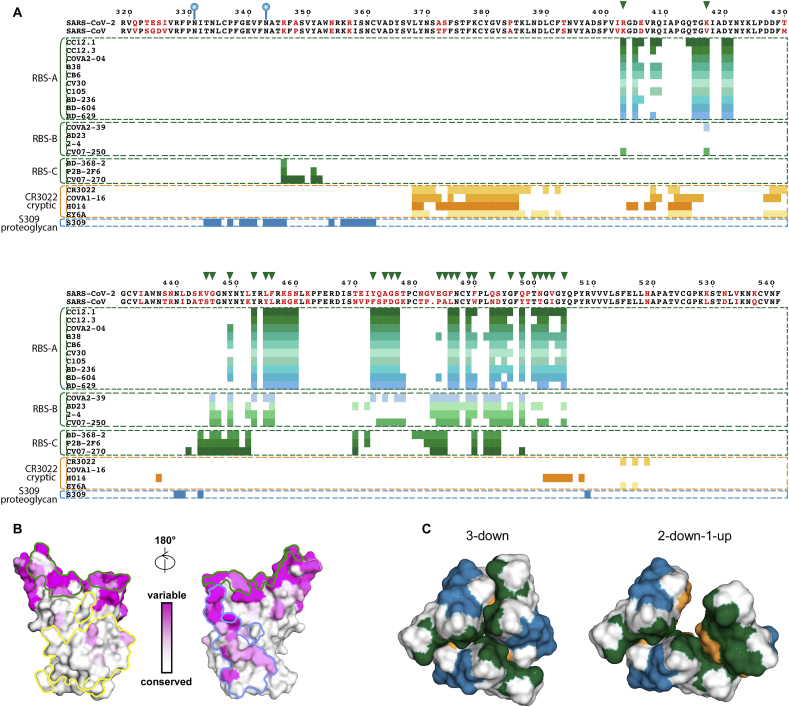
**Sequence conservation of the receptor-binding, CR3022, and S309 sites and epitope characterization. (A)** Sequence alignment between RBDs of SARS-CoV-2 and SARS-CoV, with non-conserved residues highlighted in red. Residue numbers corresponding to SARS-CoV-2 RBD are labelled every ten residues above the sequence panel and the two N-glycosylation sites by blue balloons. Clusters of antibodies bound to different epitopes are shown between the colored dashed lines, with epitope residues represented as colored bars under the sequence alignment panel. Interactions between the RBD and its ligands including antibodies and ACE2 were analyzed using PISA program [83] with the same PDB accession codes as in Fig. 3 for RBS antibodies and 6W41, 7JMW, 7CAH, 6ZCZ, 6WPS, 6M0J for CR3022, COVA1-16, H104, EY6A, S309, ACE2 in complex with RBD, respectively. The analysis here uses buried surface (BSA > 0 Å^2^) as the criterion rather than contact residues for defining the epitope. Epitopes of RBS antibodies are shown in various shades of green and light blue, CR3022 cryptic site antibodies in various shades of yellow and orange, and S309 proteoglycan site antibody in dark blue. ACE2-binding residues are represented by green triangles on top of the sequence alignment. **(B)** Sequence conservation of the RBD [from white (high) to magenta (low)] among 17 SARS-like coronaviruses^3^ is highlighted on the RBD structure with the receptor-binding site outlined in green, CR3022 site in yellow, and S309 site in blue. **(C)** Spatial relationship among the epitope sites. To simplify the view, only epitopes of CC12.1 (green), COVA1-16 (orange) and S309 (blue) are selected to represent the RBD epitopes of the RBS, CR3022 cryptic site, and S309 proteoglycan site, respectively. Cryo-EM structures of the SARS-CoV-2 S protein (PDB ID 6VXX and 6VYB) are used to represent the 3-down and 2-down-1-up structures of the RBDs.

The CR3022 site has now been found to be a target of cross-neutralizing antibodies against SARS-CoV-2, such as COVA1-16 [18,32], H014 [45], EY6A [14], and ADI-56046 [44]. COVA1-16 was isolated from a COVID-19 convalescent patient with a high affinity against the SARS-CoV-2 RBD [18,32]. Its epitope is highly similar to CR3022 as 17 of 25 epitope residues are shared with the CR3022 epitope (Fig. 6A) [32]. In contrast to CR3022, COVA1-16 shows potent neutralizing activity against SARS-CoV-2 and moderate activity against SARS-CoV pseudoviruses (IC_50_: 0.02 and 29 μg/ml, respectively, Table 2) [32]. Structural analysis of COVA1-16/RBD complex shows that the epitope of COVA1-16 extends further towards the RBS but does not overlap with the ACE2 binding site. Nevertheless, its angle of approach to the RBD causes a steric clash with ACE2. Competition assays have confirmed block of ACE2 binding by COVA1-16. These findings then suggest a possible explanation for the neutralization activity of COVA1-16 against SARS-CoV-2 and SARS-CoV that would differ from CR3022, which does not block ACE2 receptor binding (Fig. 6A) [31,32].

**Table 2:** Summary of affinity and neutralization data for cross-neutralization antibodies discussed in this review.

IgG	SARS-CoV-2			SARS-CoV	
	*K*_*D*_ (nM)	Authentic IC_50_ (μg/ml)	Pseudovirus IC_50_ (μg/ml)	*K*_*D*_ (nM)	Pseudovirus IC_50_ (μg/ml)
CR3022 [31]	<0.1	>400	N.D.	<0.1	50–100 (authentic virus)
COVA1-16 [18,32]	0.2	0.75	0.02	125	29
H014 [45]	0.096	38.1	0.4^a^	0.37	0.1^a^
EY6A [14]	8	0.07, 20^b^	N.D.	N.D.	N.D.
S309 [33]	<0.001	0.079	0.5^a^	<0.001	0.12–0.18

N.D. denotes not determined.

a Data converted from original data when reported in other units.

b Two IC_50_ values from different neutralization experiments are from Ref. [14].

Another cross-neutralizing antibody H014 is a humanized antibody that originally isolated from a mouse immunized with recombinant SARS-CoV-2 RBD using phage display [45]. With strong binding affinity to both SARS-CoV-2 and SARS-CoV RBDs and relatively high neutralization activity against both pseudotyped viruses (IC_50_: ∼0.4 and 0.1 μg/ml, Table 2), H014 sterically competes with ACE2 for RBD binding although its epitope also does not overlap with ACE2-binding site, as visualized in the cryo-EM structure of H014 in complex with the SARS-CoV-2 spike [45]. Thus, both COVA1-16 and H014 with similar but not identical epitopes show steric hindrance of ACE2 binding as well as cross-neutralization against SARS-CoV-2 and SARS-CoV.

However, not all cross-neutralizing antibodies targeting this site have established competition with ACE2. EY6A was isolated from a convalescent COVID-19 patient [14]. Structural analysis revealed that EY6A also binds to this cryptic epitope, but has no steric hindrance to ACE2 binding except for a potential clash with glycans at N322 and N546 of ACE2, as suggested [14]. Although this potential clash with EY6A could not prevent receptor binding to the RBD, EY6A substantially increased the dissociation rate between ACE2 and RBD, which may in part explain its moderate neutralization activity (Table 2).

Although the CR3022 cryptic site is not accessible when RBD is in the “down” state, a series of cryo-electron microscopy (cryo-EM) and cryo-electron tomography (cryo-ET) studies have observed that RBDs have a tendency to adopt a variety of conformations including tilting and twisting of the RBD with different ligands (ACE2, antibodies) versus the unliganded configuration [36,42,[45], [46], [47], [48], [49], [50]]. This conformational flexibility seems to be an intrinsic nature of the RBD on the spike protein that might aid in scanning for the human receptor ACE2 on the appropriate cell surface. Thus, the cryptic CR3022 site on the RBD can also be at least transiently exposed on the spike surface to engage these CR3022-like antibodies.

The high sequence conservation at the CR3022 site can be explained by structural constraints in the evolution of SARS-related viruses. Structural analysis showed that this CR3022 cryptic site is involved in the RBD-RBD interface in the down conformation and also makes contacts to the S2 subdomain within the trimeric spike protein, which may exert a negative selection pressure on the CR3022 site (Fig. 6B). Another possibility is that the CR3022 site may have more limited accessibility to antibodies since it is mostly solvent inaccessible. As a result, positive selection from immune pressure may be minimized in natural infection so far. Notwithstanding, although cross-neutralizing antibodies are uncommon in convalescent COVID-19 patients [17,18,21,51], structure-based vaccine design may enhance the antibody response to this highly conserved and vulnerable site.

## 5 Cross-neutralizing antibodies: S309 proteoglycan site

Unlike the highly conserved cryptic site targeted by cross-neutralizing antibodies, the N343 glycan site of the SARS-CoV-2 spike (corresponding to N330 of the SARS-CoV spike, Fig. 6A) is less conserved than the CR3022 site (Fig. 6B) and exposed to solvent regardless of the “up” or “down” state of the RBD (Fig. 6C). S309 is a cross-neutralizing antibody that was isolated from a preserved blood sample of a patient who survived SARS-CoV infection 17 years ago, and is the first antibody that was identified to bind to this site [33]. Structural analysis showed that most of S309 epitope residues are nevertheless conserved within sarbecoviruses. The N343 glycan is unusual for SARS-CoV-2 antibodies so far in that it seems to facilitate binding due to hydrogen bonding of its core fucose moiety to the RBD. The binding of S309 also seems to not depend on the state of the SARS-CoV-2 spike since S309 can bind in “up” and “down” RBD conformations as revealed in the spike/Fab complex cryo-EM structure [33]. S309 also does not compete with ACE2 binding as shown by the structure and competitive binding assays. Nevertheless, S309 neutralizes MLV pseudotyped SARS-CoV-2 and SARS-CoV viruses with relatively high potency (IC_50_: 0.6 and 0.5 μg/ml, Table 2), although not to the same extent as the most potent RBD antibodies. S309 also exhibits antibody-dependent cellular cytotoxicity (ADCC) and antibody-dependent cellular phagocytosis (ADCP) that can contribute to therapeutic utility, although the precise mechanism of its neutralization in cell-based pseudovirus assays remains unclear. Two important questions that should be answered soon are whether SARS-CoV-2 infection can also elicit cross-neutralizing antibodies to this site and whether they share a similar neutralization mechanism.

Other cross-neutralizing antibodies also bind to the RBDs of SARS-CoV-2 and SARS-CoV. However, detailed high-resolution structures are not available for these antibodies in complex with SARS-CoV-2 spike or RBD, such as ADI-56046 [44], S315 [33], S304 [33], 47D11 [43], CC6.33 [21] and CV38-142 [6] as well as nanobodies such as VHH-72 [52]. It will be exciting to decipher the structural basis of their cross-neutralizing activity so we can add such insights to the growing arsenal of cross-neutralizing antibodies to SARS-like viruses (sarbecoviruses).

## 6 Importance of the avidity effect in antibody neutralization

The “Y” shape antibody IgG molecule has two identical antigen-binding sites in each of the Fab arms. As a result, an IgG is capable of bivalent binding, which in turn can lead to an avidity effect over an Fab. The importance of avidity in antibody neutralization has been observed for viruses that have a relatively high density of their viral surface glycoproteins [53], such as respiratory syncytial virus (RSV) [54] and influenza virus [55,56]. Sufficient density of glycoproteins on the viral surface could enable antibodies to bind bivalently to two glycoproteins (i.e. crosslinking) [53]. In contrast, an avidity effect is rarely observed for viruses with a low density of viral surface glycoproteins, such as human immunodeficiency virus (HIV) [53]. It appears that the SARS-CoV-2 spike density is somewhat intermediate, as observed on virions by cryo-ET [57].

One way to assess whether avidity plays a role in virus neutralization is to compare the neutralization potencies of IgG and Fab. If there is no avidity effect, the neutralization potencies of IgG and Fab would be the same. Otherwise, IgG should exhibit higher neutralization as compared to the Fab. The importance of avidity in SARS-CoV-2 neutralizing antibodies has indeed been demonstrated using purified polyclonal IgGs and Fabs from SARS-CoV-2 convalescent plasma samples [30]. Specifically, Barnes et al. observed that polyclonal IgGs have a higher neutralization potency than polyclonal Fabs derived from the same convalescent plasma sample [30]. A similar observation has also been made for a monoclonal antibody in our recent study, where a cross-neutralizing RBD-targeting antibody COVA1-16 only exhibits neutralizing activity as an IgG, but not as an Fab [32]. The average distance between prefusion S trimer of SARS-CoV-2 is around 150 Å [57], which is similar to other coronaviruses [58] and comparable to the distance spanning the tip of two Fabs on an IgG [53]. Consequently, the apparent avidity of SARS-CoV-2 neutralizing antibodies can be achieved through cross-linking of two different S trimers on the virus surface. Interestingly, a SARS-CoV-2 neutralizing antibody 5A6 that was discovered from a naïve human antibody library is able to bind bivalently to two RBDs within the same spike protein, as shown by cryo-EM [59], suggesting that avidity in SARS-CoV-2 neutralizing antibodies can also be achieved through intra-spike bivalent binding. Nevertheless, structural modelling suggests that the extent of the avidity effect depends on the approach angle and binding orientation of the specified antibody [30]. Our recent study showed that the neutralizing activity of CR3022 Fab and IgG are almost identical [42], suggesting avidity is not universal to all SARS-CoV-2 neutralizing antibodies to the S protein. Future studies will need to identify the properties of antibodies (e.g. epitopes, angle of approach etc.) that correlate with avidity enhancement. Given that the binding and neutralization of Fabs is weak for several antibodies that are highly potent as IgGs, then avidity is clearly an advantage in these SARS-CoV-2 antibodies and may compensate to some extent for the low SHM found in most of these antibodies.

Several studies on RBD-targeting single domain antibodies have also taken advantage of avidity through dimerization or multimerization to enhance neutralization [[60], [61], [62]]. Similarly, dimeric ACE2 is two orders of magnitude more potent than monomeric ACE2, indicating a strong avidity effect [63]. Although it is unclear whether these avidity effects in general are due to inter-spike or intra-spike binding, these results demonstrated that avidity enhancement can be crucial for SARS-CoV-2 therapeutic design against the S protein and for assessing antibody neutralization potency.

## 7 Emergence of antibody escape mutants

**Fig. 7:**
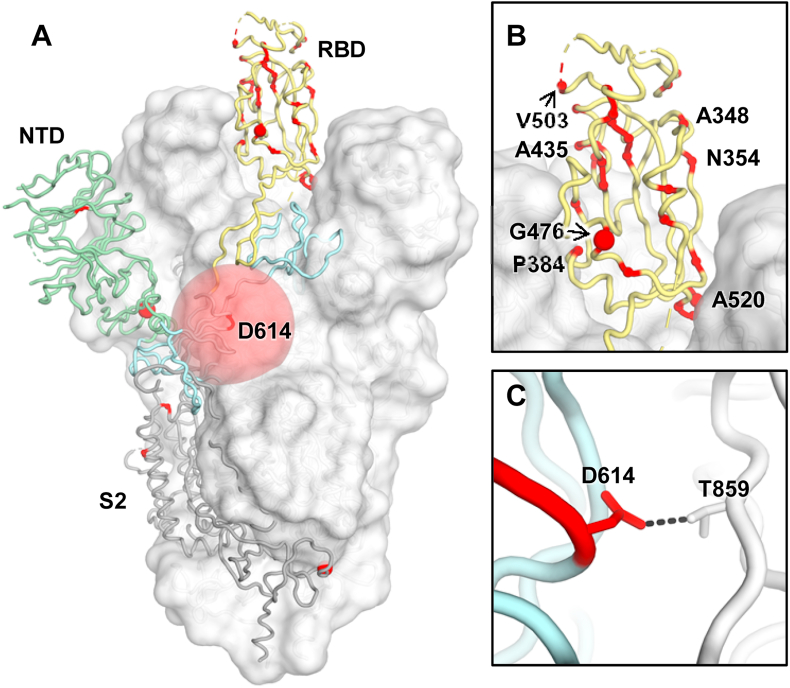
**Natural variants of SARS-CoV-2 spike protein.** Natural variants are highlighted in red, with sphere sizes corresponding to the frequency of each variant as reported in reference [72]. For clarity, only one protomer is represented in tubes and the other two are shown in a white surface (PDB ID: 6VSB^4^) [46]. **(A)** For the S1 domain, RBD (in an up conformation) and NTD are highlighted in yellow and green tubes, respectively, The S1 SD domains and S2 shown in light blue and grey, respectively. The rest of the S trimer is shown in a white surface representation. **(B)** Natural variants on the RBD are highlighted. **(C)** The major natural variant D614G is highlighted in red. D614 (red) can form a hydrogen bond with T859 (white) in an adjacent protomer, but G614 cannot form this H bond.

As SARS-CoV-2 continues to circulate in humans, many natural mutations on the S protein have started to emerge (Fig. 7). D614G is perhaps the most well-known mutation, due to its prevalence [64] as well as its increased transmissibility and infectivity [[65], [66], [67], [68], [69], [70]]. Nevertheless, recent studies have indicated that D614G does not change the antigenicity of the S protein [68], and may even increase the susceptibility of the SARS-CoV-2 to antibody neutralization [66,71]. Therefore, it seems like the main selection pressure on the evolution of the SARS-CoV-2 S protein has been to increase transmissibility and infectivity. Selection pressure from herd immunity may be weak at present since most of the population remains uninfected. However, some minor natural variants with an occurrence frequency of <1%, including A475V, L452R, V483A, F490L, and H519P on the RBD, have been shown to alter antigenicity, as assessed by both monoclonal antibodies and convalescent sera [72]. Escape mutations to monoclonal antibodies have also been identified by in vitro selection [73,74]. These observations suggest that SARS-CoV-2 could undergo antigenic drift in the future if it becomes a seasonal virus.

A well-designed antibody cocktail can also potentially limit escape, as demonstrated by studies in Zika virus, Ebola virus, and hepatitis B virus [[75], [76], [77]]. Similarly, a combination of neutralizing antibodies that bind to the different epitopes that are currently known on SARS-CoV-2 RBD, and potentially other non-RBD epitopes, can minimize the emergence of escape mutants [74]. In fact, even when an antibody cocktail consists only of antibodies that compete for binding to SARS-CoV-2 RBD, the emergence of escape mutants can still be minimized as long as escape mutations to each individual antibody in the cocktail are different [73]. It appears that the known epitopes are sufficiently distinct, even to RBS, for that to be a possibility. Several COVID-19 vaccines are currently in phase 3 clinical trials and may be approved for public use in the foreseeable future. Once COVID-19 vaccines are distributed globally, herd immunity can build up rapidly and may impose evolutionary pressure on SARS-CoV-2. Thus, now is the time to intensively investigate the potential for vaccine escape [[72], [73], [74]], which will be informative for development next-generation COVID-19 vaccines.

## 8 Conclusion

Since the publication of the first structure of antibody in complex with SARS-CoV-2 RBD half a year ago [31], many other antibody structures that target the SARS-CoV-2 RBD have now been determined. This compendium of structures has provided important insights into the antigenicity and main sites of vulnerability on SARS-CoV-2. However, it remains to be seen whether all of the epitopes on the RBD have yet been identified and whether regions that are not currently known to be targeted by antibodies are truly non-immunogenic or elicit antibodies less frequently. Since most neutralizing antibodies to SARS-CoV-2 target the immunodominant RBD, structural studies have been focused so far on RBD-targeting antibodies. Nevertheless, neutralization antibodies to the NTD have also been discovered, some of which have similar neutralization potency as the RBD-targeting antibodies [11,15]. Compared to RBD-targeting antibodies, NTD-targeting antibodies are much less well-characterized as well as antibodies to the S2 domain and quaternary antibodies [15,18,21,35]. As the COVID-19 pandemic is unlikely to be resolved in the near future and other coronavirus strains remain a potential pandemic threat, structural characterization of SARS-CoV-2 antibodies will continue to provide important insights into vaccine and therapeutic development, as demonstrated for other viruses such as influenza virus, RSV, HIV and hepatitis C virus (HCV) [[78], [79], [80], [81], [82]]. It is remarkable how much structural information has already been amassed in such a relatively short time and a testament to prior investment in technologies, techniques and researchers by funding agencies and our institutions over the years for investigation of microbial pathogens.

## Declaration of competing interest

The authors declare that they have no known competing financial interests or personal relationships that could have appeared to influence the work reported in this paper.

## Footnotes

3 The 17 SARS-like coronaviruses include SARS-CoV, SARS-CoV-2, pangolin-CoV, RaTG13, WIV1, WIV16, BM48-31, GX2013, HKU3-1, ZC45, ZXC21, Longquan-140, Hub 2013, Rp3, Rs672, Rf1, and SX2013.

4 Natural variants L5F, L8V, L8W, G446V, L452R, K458R, K458 N, D467V, I468F, I468T, I472V, A475V, G476S, T478I, V483A, V483I, F490L, and P1263L are not shown as these residues are not defined in the S protein structure (PDB ID: 6VSB).

1 The IGHV3-53 and IGHV3-66 gene segments only differ in only one amino acid position (V_H_ Ile 12 for IGHV3-53 verses V_H_ Val 12 for IGHV3-66).

2 Sequence identity of the receptor-binding motifs of SARS-CoV and SARS-CoV-2 is only 57%, whereas sequence identity of the rest of the RBD is 92%.
